# Venereal Affections

**Published:** 1855-02

**Authors:** 


Venereal Affections.—

*Bubo.*—I is seldom necessary to open a bubo. Employ counter-irritation. Take a solution of nitrate of silver (one drachm to two drachms of water), with the addition of three drops of strong nitric acid. This is to be painted freely into the skin over the inflamed gland. It causes great pain and soreness, which is followed by rapid diminution of the gland. This treatment may be extended to other cases in which inflammatory deposits, whether purulent or fibrinous, require to be absorbed or diminished.

*Syphilis.*—Treat the infecting chancre, the true, firm, indurated sore, with mercury. The unindurated sore may be safely treated without mercury. In treating the former, in Paris, the proto-iodide of mercury is used, but this does not answer so well in this country as mercurial frictions, blue pill, or the grey powder.

The *Phagedænic Chancre* is treated by Ricord, by first destroying the whole of the unhealthy surface with the red hot iron, previously placing the patient under the influence of chloroform. After the sloughs have separated, the surface is covered with strips of ammoniacum and mercurial plaster, and doses of tartrate of iron are given with great success, in the less obstinate cases. The most frequent variety of sore in London is the phagedmænic, with induration. In this, the actual cautery does no good; mercury must here be given, and the system supported by bark, wine, or steel.—*Braithwait's Retrospect.*

